# Effects of Light-Emitting Diodes on the Accumulation of Glucosinolates and Phenolic Compounds in Sprouting Canola (*Brassica napus* L.)

**DOI:** 10.3390/foods8020076

**Published:** 2019-02-19

**Authors:** Chang Ha Park, Nam Su Kim, Jong Seok Park, Sook Young Lee, Jong-Won Lee, Sang Un Park

**Affiliations:** 1 Department of Crop Science, Chungnam National University, 99 Daehak-ro, Yuseong-gu, Daejeon 34134, Korea; parkch804@gmail.com (C.H.P.); kns917555@naver.com (N.S.K.); 2 Department of Horticultural Science, Chungnam National University, 99 Daehak-ro, Yuseong-gu, Daejeon 34134, Korea; jongseok@cnu.ac.kr; 3 Marine Bio Research Center, Chosun University, 61-220 Myeongsasimni, Sinji-myeon, Wando-gun, Jeollanamdo 59146, Korea; seedbank@chosun.ac.kr; 4 Department of Horticulture Environment System, Korea National College of Agriculture and Fisheries, 1515, Kongjwipatjwi-ro, Deokjin-gu, Jeonju-si, Jeollabuk-do 54874, Korea

**Keywords:** light-emitting diode, sprouting canola, sprouts, secondary metabolites

## Abstract

In this study, we investigated optimal light conditions for enhancement of the growth and accumulation of glucosinolates and phenolics in the sprouts of canola (*Brassica napus* L.). We found that the shoot lengths and fresh weights of red light-irradiated sprouts were higher than those of sprouts exposed to white, blue, and blue + red light, whereas root length was not notably different among red, blue, white, and blue + red light treatments. The accumulations of total glucosinolates in plants irradiated with white, blue, and red lights were not significantly different (19.32 ± 0.13, 20.69 ± 0.05, and 20.65 ± 1.70 mg/g dry weight (wt.), respectively). However, sprouts exposed to blue + red light contained the lowest levels of total glucosinolates (17.08 ± 0.28 mg/g dry wt.). The accumulation of total phenolic compounds was the highest in plants irradiated with blue light (3.81 ± 0.08 mg/g dry wt.), 1.33 times higher than the lowest level in plants irradiated with red light (2.87 ± 0.05 mg/g dry wt.). These results demonstrate that red light-emitting diode (LED) light is suitable for sprout growth and that blue LED light is effective in increasing the accumulation of glucosinolates and phenolics in *B. napus* sprouts.

## 1. Introduction

Plant sprouts, defined as young shoots formed from seeds, have been recognized as outstanding sources of essential and non-essential nutrients such as carbohydrates, proteins, minerals, and vitamins. Furthermore, sprouts can aid in the prevention of diverse diseases due to their biological activities (antioxidant, anticancer, antigenotoxic, and antibiotic effects), which are associated with health-maintaining compounds (glucosinolates, polyphenols, terpenes, sterols, and vitamins) [1,2]. A previous study reported that plant sprouts have considerably higher levels of certain health-protecting phytochemicals than those discovered in the mature plant [3]. In particular, *Brassica* sprouts have been consumed as raw materials and provide a variety of bioactive compounds (glucosinolates, isothiocyanates, polyphenols, anthocyanins, and carotenoids) that can contribute to the prevention and treatment of diseases [1,2,4,5,6,7,8].

Canola (*Brassica napus*), belonging to the Brassicaceae family, has been cultivated and harvested worldwide for its seeds, which can be used as a source of provender, an appetizing vegetable oil, and biodiesel [9,10]. Previous studies have reported that the seeds of canola varieties contain various hydroxycinnamic acid derivatives (*p*-coumaric, caffeic, ferulic, and sinapic acids) and that the leaves of canola contain benzoic acid derivatives (gentisic, p-hydroxybenzoic, protocatechuic, syringic, and vanillic acids). In particular, sinapic derivatives are dominant phenolic compounds in these seeds [2,11,12]. Glucosinolates are sulfur-containing bioactive compounds present in *Brassica* vegetables. Brown and Morra [13], Vierheilig et al. [14], and Yasumoto et al. [15] detected six glucosinolates (progoitrin, gluconapin, 4-hydroxyglucobrassicin, glucobrassicanapin, glucobrassicin, and gluconasturtiin) in *Brassica* vegetables, whereas Yasumoto et al. [15] reported differences in six glucosinolates in different organs of canola cv. Kirariboshi sprouts.

Glucosinolates, which contain nitrogen and sulfur structures, are a group of secondary metabolites in the Cruciferae family and can be classified into three classes (aliphatic, aromatic, and indolic glucosinolates) [16]. Recently, interest in glucosinolates has increased owing to the biocidal [17] and cancer chemopreventive activity [18] of their hydrolysis products (isothiocyanates, nitriles, thiocyanates, epithionitriles, and oxazolidines). In particular, intake of isothiocyanates from *Brassica* vegetables has been shown to reduce cancer risk [19,20].

Plant phenolic compounds are secondary metabolites ubiquitous in most higher plants and responsible for plant defense against biotic and abiotic stresses (pathogen and insect attack, excess light and ultraviolet radiation, extreme temperature, wounding, and nutrient deficiencies) [21,22]. Furthermore, dietary phenolic compounds in plant-based foods may be beneficial to human health, since such compounds have anti-human immunodeficiency virus (HIV) [21,23], antioxidant [21,24], anticancer [21,25], anti-inflammatory [21,26], anticariogenic [21,27], and cardioprotective [21,28] properties.

Light quality and intensity are crucial for plant development, morphogenesis, growth, and pigment biosynthesis [29]. Light-emitting diodes have been successfully applied to control plant growth environments due to their advantages of high-efficiency energy conversion, long lifetime, wavelength specificity, small bandwidth and volume, controllable light quality and intensity, and low-grade thermal energy output [30]. In particular, LED lights have more specific wavelengths and smaller bandwidths compared with filters and can provide a wide range of light sources for plant growth [31].

To our knowledge, there have been no previous studies on the effects of different LED light sources on secondary metabolites in *B. napus* sprouts. Therefore, the purpose of the present study was to investigate the effects of different LED light wavelengths (blue + red (470 and 660 nm) blue (470 nm), red (660 nm), or white (380 nm)) on the growth and production of glucosinolates and phenolic compounds in *B. napus* sprouts.

## 2. Materials and Methods

### 2.1. Plant Samples

Seeds of canola were obtained from Asia Seed Co., Ltd (Seoul, Korea). The seeds were soaked into tap water for 1 day, and thereafter 200 seeds were placed in plastic pots containing vermiculite soil and watered with 100 mL of tap water. Each pot was then transferred to an incubator equipped with white (wavelength, 380 nm), blue (wavelength, 470 nm), red (wavelength, 660 nm), or blue + red LEDs. Specifically, the white, red, and blue components of the LED grow light (PARUS LED Co., Cheoan, Korea) contain 14 pieces of LED, respectively. The plant growth chamber was equipped with two LED grow lights for the white, red, and blue LED treatments. On the other hand, for the blue + red treatment, the chamber was equipped with a blue and a red LED grow light, respectively. The seeds were germinated and grown under a 16-h photoperiod with high-intensity irradiation (flux rate of 50 µmol/s·m^2^) at 25 °C. After 14 days, shoot length, root length, and fresh weight were measured and the plant tissues were harvested in liquid nitrogen. The frozen samples were then lyophilized and ground to fine powders for further HPLC analysis.

### 2.2. Extraction of Glucosinolates from Brassica napus and HPLC Analysis

Glucosinolate extraction and desulfation were performed using previously described procedures [32,33,34]. In brief, a mini-column, packed with DEAE-Sephadex A-25 (H^+^ form by 0.5 M sodium acetate, approximately 40 mg dry wt.), was prepared for glucosinolate extraction. Methanol (MeOH: 70% v/v) was boiled to 70 °C and 1.5 ml of the boiled MeOH was placed in a tube containing 100 mg of dried sprout powder. The mixture was incubated in a water bath at 70 °C for 5 min for endomyrosinase inactivation. After centrifugation at 12,000 × *g* for 15 min at 4 °C, the supernatant was transferred to a new tube. The remaining sludge was re-extracted a further two times in the same manner, and the collected supernatants were combined. The crude extract was loaded onto a mini-column and desulfated by the addition of aryl sulfatase solution (75 μL, 29 units) to the column. The desulfation reaction was carried out overnight at ambient temperature, and then 0.5 mL of HPLC-grade water was used for elution of desulfo-glucosinolates. The solution was then filtered into a vial through a 0.45-μm syringe filter. The LC conditions used followed those established in a previous study [34]. Desulfoglucosinolates were quantified according to their response factor, HPLC area, and an external sinigrin standard. The values represent the means ± standard deviation of three biological replicates.

### 2.3. Extraction of Phenylpropanoids from Brassica napus and HPLC Analysis

Samples of dried sprout powder (100 mg) were extracted with 80% (v/v) methanol and then sonicated for 1 h with vortexing every 20 min during the incubation. After centrifugation at 12,000 rpm for 15 min, crude extracts were transferred to a new tube. The remaining sludge was re-extracted a further two times in the same manner. The collected solution was filtered through 0.45-μm filters for subsequent HPLC analysis. The LC conditions used followed those established in a previous study [35]. Identification and quantification of phenolic compounds were carried out by comparison of retention times and spike tests, and corresponding calibration curves. The values represent the means ± standard deviation of three biological replicates.

### 2.4. Statistical Analysis

Growth and HPLC data were statistically analyzed with Duncan’s multiple range test at *p* < 0.05 using Statistical Analysis System software (SAS, system 9.4, 2013; SAS Institute, Inc., Cary, NC, USA). Reported values represent the means ± standard deviation of three biological replicates.

## 3. Results

### 3.1. Sprout Length and Fresh Weight

**Figure 1:**
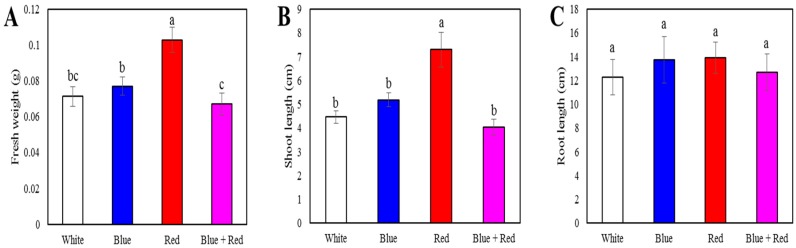
Growth of *Brassica napus* sprouts grown under different light-emitting diode (LED) lights (blue, white, red, and blue + red): (**A**) shoot length; (**B**) root length; and (**C**) fresh weight from 14 days. Each value is the mean of three biological replicates, and error bars indicate the standard deviation (SD). Different letters above bars indicate a significant difference (*p* < 0.05).

Different LEDs had a considerable effect on the shoot length and fresh weight of sprouts, with those of red light-irradiated sprouts being significantly higher than those of sprouts exposed to other LEDs (Figure 1). Specifically, the shoot lengths of red light-irradiated sprouts were 1.64-, 1.41-, and 1.81-fold higher and the fresh weights 1.44-, 1.34-, and 1.54-fold higher than those of spouts exposed to white, blue, and blue + red LEDs, respectively. In contrast, root length was not significantly different among the red, blue, white, and blue + red LED treatments.

### 3.2. Accumulation of Glucosinolates in Sprouts

Glucosinolates were analyzed in *B. napus* sprouts grown under LED irradiation (Table 1). A total of 16 glucosinolates (one aromatic glucosinolate (gluconasturtiin), four indolic glucosinolates (glucobrassicin, 4-methoxyglucobrassicin, 4-hydroxyglucobrassicin, and neoglucobrassicin), and 11 aliphatic glucosinolates (sinigrin, glucoiberin, glucobrassicanapin, glucoraphanin, gluconapoleiferin, glucoalyssin, gluconapin, glucoerucin, glucoberteroin, glucoraphasatin, and progoitrin)) were detected and quantified in the sprouts of *B. napus* through comparison of retention times, HPLC areas, and response factors with respect to those of an external standard. The levels of total glucosinolates in the canola seedlings irradiated with the white, blue, and red LEDs were not significantly different (19.32 ± 0.13, 20.69 ± 0.05, and 20.65 ± 1.70 mg/g dry wt., respectively). Seedlings exposed to the blue + red LED light contained the lowest levels of total glucosinolates (17.08 ± 0.28 mg/g dry wt.). Among the individual glucosinolates, the levels of sinigrin, glucobrassicin, and 4-methoxy glucobrassicin were higher in sprouts exposed to red LED light, whereas seedlings grown under blue LED light contained the highest levels of glucoraphanin. Furthermore, higher levels of glucoalyssin and gluconapin were obtained in sprouts exposed to white and blue LED light.

### 3.3. Accumulation of Phenolics in Sprouts

A total of eight phenolic compounds (four phenolic acids (chlorogenic, caffeic, sinapic, and benzoic acid) and four flavonoids (rutin, catechin, epicatechin, and quercetin)) were detected and quantified in the seedlings of *B. napus* through HPLC comparison of retention times, spike tests, and external standard calibration curves (Table 2). The highest level of total phenolic compounds was obtained in blue light-radiated sprouts (3.81 ± 0.08 mg/g dry wt.), which was 1.33 times higher than the lowest level obtained from red light-radiated sprouts (2.87 ± 0.05 mg/g dry wt.). Notably, the sprouts exposed to blue LED lights showed the highest levels of benzoic acid, (+)-catechin, caffeic acid, and (−)-epicatechin. In contrast, the highest levels of sinapic acid and rutin were obtained in red light-irradiated sprouts, whereas the levels of chlorogenic acid and quercetin were higher in seedlings exposed to white and blue LED lights.

## 4. Discussion

Plant development, morphogenesis, growth, and secondary metabolite synthesis are significantly affected by light quality and intensity [29,36]. In the current study, red LED light was found to be suitable for sprout growth. Our findings are consistent with those obtained in previous studies. McNellis and Deng [37], for example, demonstrated that red light induces cotyledon expansion and hypocotyl elongation and that blue light induces cotyledon expansion and suppresses hypocotyl elongation in *Arabidopsis* seedlings. Similarly, Miyashita et al. [38] reported that blue light inhibited stalk elongation, whereas red light significantly enhanced elongation in pelargonium plantlets. Furthermore, red light irradiation for 4 days has been shown to increase the leaf area and stem length of pea seedlings compared with seedlings exposed to white light [39], and Thew et al. [40] reported that red LED light irradiation promoted significant increases in the shoot length and fresh weight of *Fagopyrum tataricum* sprouts compared with blue and white LED lights.

*Brassica* vegetables are a rich source of plant phenolics and glucosinolates. In the current study, we detected and quantified 16 glucosinolates and eight phenolic compounds in canola sprouts irradiated with different LEDs. These results are consistent with those of previous studies that have analyzed progoitrin, gluconapin, 4-hydroxyglucobrassicin, glucobrassicanapin, glucobrassicin, and gluconasturtiin in canola seed [13,14,15], and glucoiberin, progoitrin, sinigrin, glucoraphanin, gluconapoleiferin, gluconapin, 4-hydroxiglucobrassicin, glucobrassicanapin, glucobrassicin, gluconasturtiin, and neoglucobrassicin in leaf rape (*B. napus* var. *pabularia*) [41]. Furthermore, quercetin, (-)-epicatechin, sinapic acid, and caffeic acid have previously been identified in the seeds of *B. napus* [42].

According to the results of our phenolic compound analyses, irradiation with blue LEDs improves the production of most phenolics, including (+)-catechin, caffeic acid, and (–)-epicatechin. With respect to glucosinolate contents of the canola sprouts irradiated with different LEDs, sprouts grown under blue, red, and white LEDs showed higher levels of glucosinolates compared with blue + red LEDs. These results are consistent with the findings of previous studies. Kim et al. [6], for example, reported that exposure to blue LED light promoted a high production of most phenolics in Chinese cabbage seedlings after irradiation for 12 days, whereas Ghimire et al. [43] reported a marked increase in the accumulation of phenolics in ginseng adventitious roots. Similarly, Thew et al. [40] demonstrated that exposure to blue LED light irradiation induced upregulated phenylpropanoid biosynthesis in tartary buckwheat sprouts and production of phenolics in cowpea sprouts [44]. Additionally, exposure to blue LED light enhanced production of phenolic acids in the shoot cultures of *Aronia melanocarpa*, *Aronia arbutifolia*, and *Aronia prunifolia* [45] as well as chlorogenic acid in callus of *Peucedanum japonicum* Thunb. [46] and total phenolic contents in callus of *Ocimum bassilicum* [47].

Furthermore, exposure to blue LED light has been shown to lead to a pronounced enhancement of carotenoid biosynthesis in citrus juice sacs in vitro [48] and in the Chinese skullcap callus [49], as well as the enhanced production of glucosinolates in broccoli sprouts, compared with blue + red LED treatment [50]. Red LEDs have been reported to promote higher levels of phenolic compounds in *Myrtus communis* L. in vitro [51] and carotenoid synthesis in the flavedo of citrus fruits [52], whereas white LEDs have been found to enhance carotenoid production in the sprouts of tartary buckwheat [53]. Moreover, is has been demonstrated that the production of phenolics does not differ significantly in sprouts of common buckwheat (cv. Kitawase) and tartary buckwheat (cv. Hokkai T8) exposed to blue, red, and blue + red LEDs [54], whereas fluorescent lights have been reported to increase the levels of ginsenoside-Rg1 and ginsenoside-Rb1 in *Panax vietnamensis* plantlets relative to irradiation with diverse types of LEDs [55]. In contrast, compared with light exposure, dark conditions have been found to decrease glucosinolate biosynthesis in Chinese cabbage seedlings [56].

On the basis of the findings of the present and previous studies, it appears that the influence of different light sources and wavelengths on the production of natural products might be dependent on plant species, cells, tissues, and organs. With respect to *B. napus* sprouts, however, the results of the present study indicate that blue LED lights are the optimal light source for the production of glucosinolates and phenolics.

## Figures and Tables

**Table 1:** The accumulation of glucosinolates (mg/g dry wt.) in *Brassica napus* sprouts grown under different LED lights.

Glucosinolate	White	Blue	Red	Blue + Red
Glucoiberin	0.15 ± 0.01 a ^1^	0.14 ± 0.03 a	0.12 ± 0.01 a	0.13 ± 0.01 a
Progoitrin	9.38 ± 0.12 b	10.68 ± 0.16 a	10.01 ± 1.07 ab	7.70 ± 0.17 c
Glucoraphanin	0.21 ± 0.01 b	0.25 ± 0.01 a	0.22 ± 0.03 b	0.20 ± 0.00 b
Sinigrin	1.04 ± 0.04 b	0.82 ± 0.00 c	1.60 ± 0.14 a	1.00 ± 0.02 b
Glucoalyssin	0.07 ± 0.01 a	0.07 ± 0.00 a	0.05 ± 0.01 b	0.05 ± 0.00 b
Gluconapoleiferin	0.36 ± 0.01 ab	0.39 ± 0.03 a	0.34 ± 0.05 ab	0.32 ± 0.02 b
Gluconapin	1.56 ± 0.04 a	1.58 ± 0.02 a	1.43 ± 0.13 b	1.18 ± 0.01 c
4-Hydroxy glucobrassicin	1.89 ± 0.07 a	1.78 ± 0.12 a	1.58 ± 0.06 b	1.91 ± 0.05 a
Glucobrassicanapin	0.14 ± 0.00 a	0.13 ± 0.00 a	0.12 ± 0.01 a	0.11 ± 0.00 b
Glucoerucin	0.03 ± 0.00 ab	0.04 ± 0.00 a	0.03 ± 0.00 b	0.05 ± 0.01 a
Glucoraphasatin	0.03 ± 0.00 a	0.04 ± 0.00 a	0.04 ± 0.00 a	0.04 ± 0.00 a
Glucobrassicin	1.26 ± 0.03 b	1.07 ± 0.04 c	1.36 ± 0.04 a	1.29 ± 0.02 b
4-Methoxy glucobrassicin	0.88 ± 0.03 c	0.96 ± 0.05 b	1.05 ± 0.02 a	0.91 ± 0.02 c
Glucoberteroin	0.03 ± 0.00 a	0.03 ± 0.00 a	0.04 ± 0.01 a	0.03 ± 0.00 a
Gluconasturtiin	0.16 ± 0.00 ab	0.15 ± 0.01 ab	0.17 ± 0.02 a	0.14 ± 0.00 b
Neoglucobrassicin	2.12 ± 0.10 b	2.55 ± 0.01 a	2.54 ± 0.17 a	2.07 ± 0.04 b
Total	19.32 ± 0.13 a	20.69 ± 0.05 a	20.65 ± 1.70 a	17.08 ± 0.28 b

^1^ Different letters in the same row indicate a significant difference (*p* < 0.05).

**Table 2:** The accumulation of phenolics (mg/g dry wt.) in *Brassica napus* sprouts grown under different LED lights.

Class	Compound	White	Blue	Red	Blue + Red
Phenolic acid	Caffeic acid	0.41 ± 0.01 c	0.55 ± 0.01 a	0.40 ± 0.02 c	0.46 ± 0.02 b
	Chlorogenic acid	0.14 ± 0.01 a	0.15 ± 0.01 a	0.07 ± 0.00 b	0.15 ± 0.04 a
	Sinapic acid	0.14 ± 0.00 a	0.08 ± 0.01 b	0.10 ± 0.01 b	0.09 ± 0.01 b
Catechin	(-)-Epicatechin	1.58 ± 0.07 b	1.77 ± 0.05 a	1.26 ± 0.04 c	1.47 ± 0.11 b
	(+)-Catechin	0.44 ± 0.00 b ^1^	0.65 ± 0.01 a	0.49 ± 0.02 b	0.44 ± 0.07 b
Organic acid	Benzoic acid	ND ^2^	0.09 ± 0.01	ND	ND
Flavonoid	Rutin	0.30 ± 0.00 a	0.23 ± 0.00 d	0.28 ± 0.01 b	0.25 ± 0.01 c
	Quercetin	0.29 ± 0.00 a	0.29 ± 0.00 a	0.28 ± 0.00 b	0.28 ± 0.00 b
	Total	3.29 ± 0.04 b	3.81 ± 0.08 a	2.87 ± 0.05 d	3.14 ± 0.04 c

^1^ Different letters in the same row indicate a significant difference (*p* < 0.05). ^2^ ND, not detected.
